# Molecular architecture of the PBP2–MreC core bacterial cell wall synthesis complex

**DOI:** 10.1038/s41467-017-00783-2

**Published:** 2017-10-03

**Authors:** Carlos Contreras-Martel, Alexandre Martins, Chantal Ecobichon, Daniel Maragno Trindade, Pierre-Jean Matteï, Samia Hicham, Pierre Hardouin, Meriem El Ghachi, Ivo G. Boneca, Andréa Dessen

**Affiliations:** 1 Univ. Grenoble Alpes, CNRS, CEA, Institut de Biologie Structurale (IBS), Bacterial Pathogenesis Group, F-38000 Grenoble, France; 2 Institut Pasteur, Unit of Biology and Genetics of the Bacterial Cell Wall, F-75015 Paris, France; 3 INSERM, Groupe Avenir, F-75015 Paris, France; 4 Brazilian Biosciences National Laboratory (LNBio), CNPEM, Campinas, São Paulo, 13084-971 Brazil

## Abstract

Bacterial cell wall biosynthesis is an essential process that requires the coordinated activity of peptidoglycan biosynthesis enzymes within multi-protein complexes involved in cell division (the “divisome”) and lateral wall growth (the “elongasome”). MreC is a structural protein that serves as a platform during wall elongation, scaffolding other essential peptidoglycan biosynthesis macromolecules, such as penicillin-binding proteins. Despite the importance of these multi-partite complexes, details of their architecture have remained elusive due to the transitory nature of their interactions. Here, we present the crystal structures of the soluble PBP2:MreC core elongasome complex from *Helicobacter pylori*, and of uncomplexed PBP2. PBP2 recognizes the two-winged MreC molecule upon opening of its N-terminal region, revealing a hydrophobic zipper that serves as binding platform. The PBP2:MreC interface is essential both for protein recognition in vitro and maintenance of bacterial shape and growth. This work allows visualization as to how peptidoglycan machinery proteins are scaffolded, revealing interaction regions that could be targeted by tailored inhibitors.

## Abstract (web summary)

Bacterial wall biosynthesis is a complex process that requires the coordination of multiple enzymes. Here, the authors structurally characterize the PBP2:MreC complex involved in peptidoglycan elongation and cross-linking, and demonstrate that its disruption leads to loss of *H. pylori* shape and inability to sustain growth.

## Introduction

The peptidoglycan (PG) is an essential component of the bacterial cell wall, and plays a key role in shape maintenance, resistance to osmotic pressure, and cell division. PG is formed by polymerized N-acetyl-glucosamine (GlcNAc) and N-acetyl-muramic acid (MurNAc) units cross-linked by stem peptides that generate a mesh-like structure that surrounds and stabilizes the entire cell. Due to the central role PG plays in bacterial survival, its biosynthetic machinery has been a prime target for antibiotic development for decades^1^. Proteins that are involved in PG biosynthesis associate in discrete multi-membered complexes that regulate cell division (the “divisome”) and cell wall elongation (the “elongasome”), and their inhibition or deregulation can lead to defects in cell shape, impaired growth, and often cell wall lysis and death^2, 3^.

Penicillin-binding proteins (PBPs) catalyze the two last reactions in PG biosynthesis (GlcNAc-MurNAc polymerization and stem peptide cross-linking, or transpeptidation). Class A enzymes can catalyze both reactions, while class B PBPs act uniquely as transpeptidases. PBPs are the targets of β-lactam antibiotics and many are involved in resistance to these antibiotics in numerous pathogens^4^. PBPs have been reported to interact with various members of both the divisome and the elongasome within the periplasm and the bacterial membrane. Within the elongasome of a variety of bacteria including *E. coli*, *Caulobacter crescentus*, *Bacillus subtilis*, and *Helicobacter pylori*, PBPs have been shown to interact with, and be recruited by, the essential protein MreC, a bitopic membrane protein that is essential for cell shape^5–13^. This process is orchestrated by the cytoplasmic actin homolog MreB, pointing to the existence of a multi-membered complex that spans cytoplasm, membrane, and periplasm. Disruption of these interactions within the elongasome leads to cell shape perturbation and eventual bacterial cell death^5–13^, indicating the importance of the tight orchestration of elongasome formation steps. In *H. pylori*, both PBP2 (class B) and MreC are essential proteins, and strains depleted for either gene lose their rod-shaped morphology, enter division arrest and become enlarged cocci^8^. MreC has thus been suggested as being a scaffold for elongasome formation, docking PBPs^13^, and generating a core complex onto which other PG-biosynthesis partners can successively tether^8^. Despite the vast amount of functional evidence of the importance of the PBP:MreC interaction for bacterial cell wall formation, the absence of structural data regarding any periplasmic cell wall-formation complex has hampered detailed studies of divisome or elongasome architecture. In addition, such complexes have been reported to only form at defined points in the cell cycle, and are thus fleeting in nature, fragile, and difficult to isolate^14^.

## Results

### PBP2 and MreC interact through a hydrophobic zipper

We have overcome these difficulties and structurally and functionally characterized the PBP2:MreC core elongasome complex from the human pathogen *H. pylori*, as well as PBP2 in its unbound form. The crystal structure of PBP2 from *H. pylori* was initially solved to 3.0 Å by molecular replacement using the structure of PBP3 from *Pseudomonas aeruginosa* ^15^ as a model. PBP2:MreC complex crystals yielded diffraction data to 2.7 Å and harbored two 1:2 PBP2:MreC complexes in the asymmetric unit. Diffraction data were phased by using our structure of PBP2 from *H. pylori* as a model in a molecular replacement experiment. Multiple rounds of manual and automatic model building, followed by molecular refinement, were required for the generation of both PBP2 and PBP2:MreC structures.

**Fig. 1:**
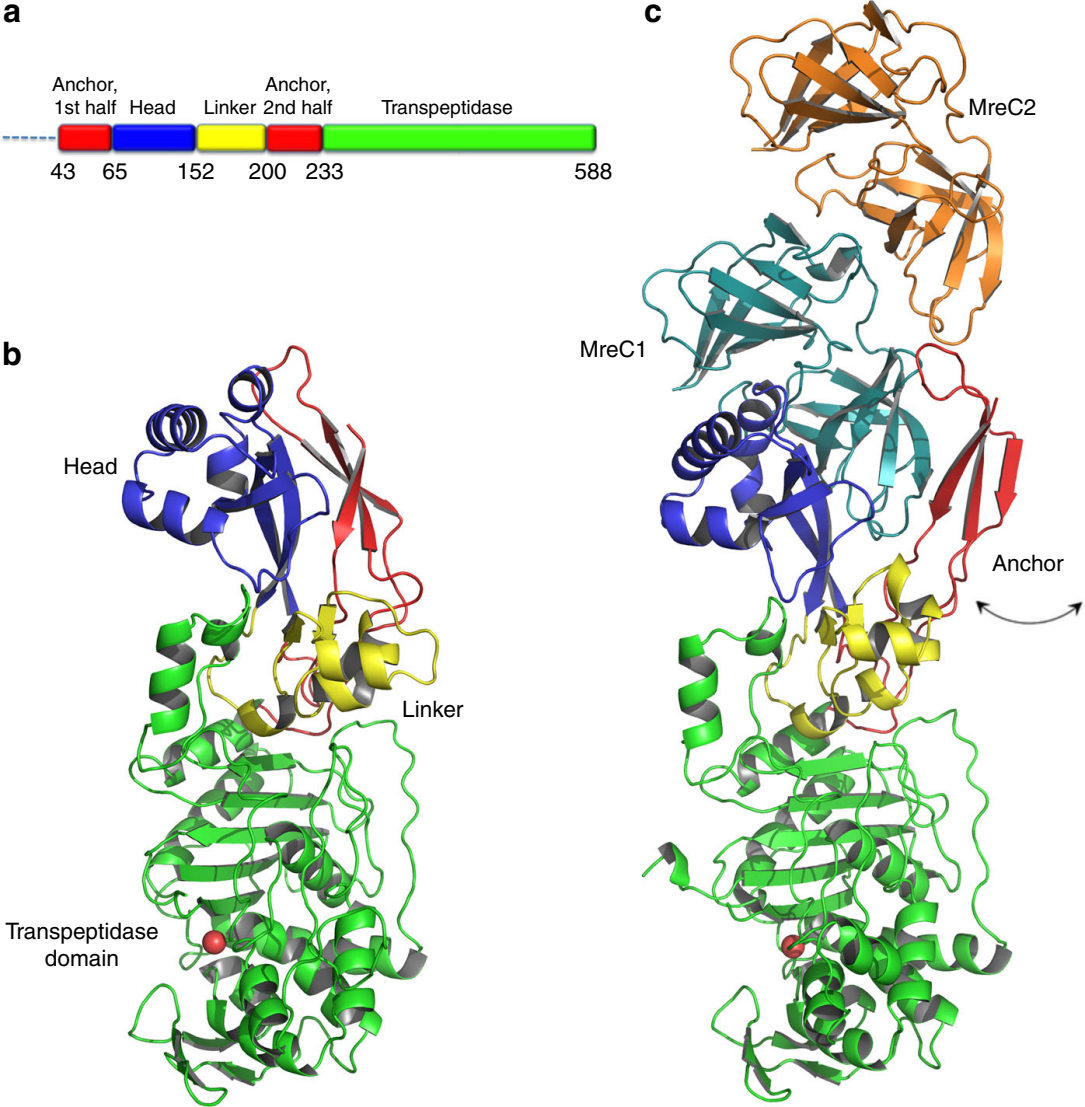
Architecture and structural arrangement of PBP2 and the PBP2:MreC complex from *H. pylori*. **a** Schematic diagram of PBP2 and the limitations of different domains. **b** PBP2 folds into anchor, head, linker, and transpeptidase domain. The anchor is “clasped” against the helical head. **c** The PBP2 anchor region sways away from the head through movement of a hinge region, exposing a previously hidden hydrophobic region that allows binding of MreC1

**Fig. 2:**
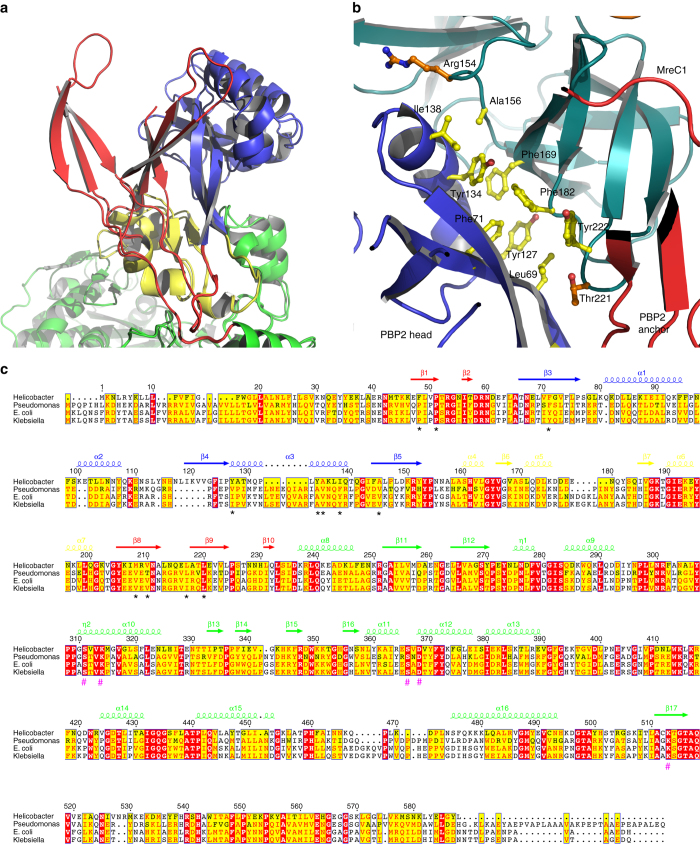
MreC binding engenders opening of the N-terminus of PBP2. **a** Overlay of MreC-bound and unbound PBP2 structures, indicating movement of the anchor region (in *red*). Opening of the N-terminal region of PBP2 exposes a hydrophobic region on the head that is complemented by a non-polar face on the surface of MreC (**b**), forming a hydrophobic zipper whose disruption hinders complex formation. **c** Structure-based sequence alignment between class B PBPs from *H. pylori* (this work), *P. aeruginosa* PAO1 G3XCV7, *E. coli* O157:H7 P0AD67, and *K. pneumoniae* A0A0W8ASI8 (UniProt codes). Head, anchor, linker, and TP are indicated in color above the sequence. Residues involved in interaction between head and anchor are indicated with *black asterisks*. Active site residues are indicated with *hash tags*. Figure generated with ESPRIPT^53^

PBP2 (residues L38–L588, thus lacking the TM helix) is a 588-residue molecule that presents the characteristic fold of class B PBPs, with an elongated N-terminal region followed by a transpeptidase domain that carries the stem peptide cross-linking site (Fig. 1 and Supplementary Fig. 1). The N-terminal region of PBP2 displays a tripartite structure: (1) a mostly helical “head” (*dark blue* in Fig. 1), (2) an “anchor” region composed of three β-strands (*red*), and (3) a helix-rich linker domain (*yellow*). The transpeptidase domain harbors the active site (Supplementary Fig. 1a) with the three conserved motifs: S–X–X–K (Ser311′–Val312′–Val313′–Lys314′), which includes the catalytic serine, located at the N-terminus of α2; S–X–N/D (Ser366′–Val367′–Asp368′), which lies between α4 and α5; and K–T/S–G (Lys513′–Thr514′–Gly515′), located at the C-terminus of β3 (PBP2 numbering indicated with a prime symbol). It is of note that a disulfide bond lies in the proximity of the active site, linking β3 to α9; a similar positioning for a disulfide bond in SpoVD (PBP2) from *B. subtilis* was recently linked to regulation of catalytic activity^16^.

In the structure of the complex, MreC folds as two tandem β-barreled domains shaped as “butterfly wings” that are highly similar to those identified in MreC variants from *Streptococcus pneumoniae* and *Listeria monocytogenes* ^17, 18^ (Fig. 1 and Supplementary Fig. 1b). It is of note that our MreC clone includes residues 37–248 (thus lacking only the short cytoplasmic and TM regions), but only residues 92–248 are visible in the structure. Two “butterflies” are present in the asymmetric unit of the complex, but only one interacts directly with PBP2, the other one interacting uniquely with its partner MreC. Thus, MreC employs two distinct surfaces for partner molecule recognition, a fact that relates directly to its biological role of scaffold protein.

**Fig. 3:**
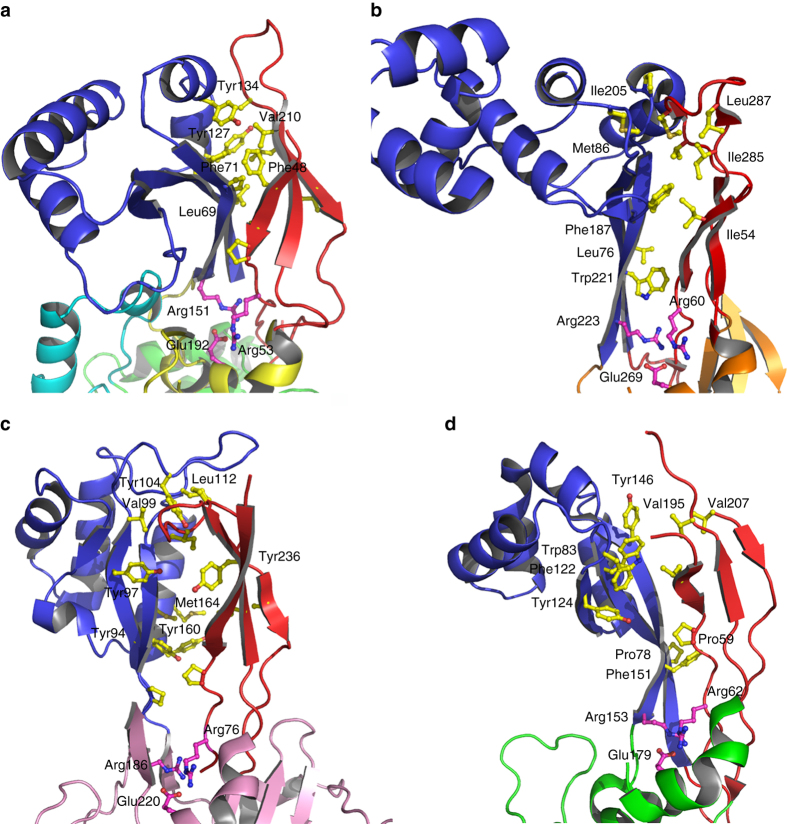
Class B PBPs from Gram-negative and Gram-positive display similarities at key interfaces. **a** PBP2 from *H. pylori*, this work, **b** PBP2b from *S. pneumoniae* (2WAD), **c** PBP2x from *S. pneumoniae* (1RP5), **d** PBP3 from *P. aeruginosa* (3PBN) harbor anchor (*red*) and head (*blue*) regions stabilized by hydrophobic interactions, which could be disrupted and reoriented upon binding to a partner PG-biosynthesis molecule (such as MreC). An Arg-Arg-Glu constellation, indicated here in *magenta*, is present in the structures of all class B PBPs solved to date, and sits at the interface between anchor and head

MreC interacts with PBP2 by inserting its C-terminal β-domain between the head and anchor regions of the PBP, forcing the anchor region to “sway” by ~15 Å away from the head (Fig. 1). The swaying movement is guaranteed by the flexibility of loops 50′–52′, 203′–204′, and 224′–227′ that act as a hinge, opening up the N-terminus into a “V” shape that accommodates MreC snugly (Fig. 2a). Direct interactions involve mostly the head region of PBP2, which recognizes MreC through a hydrophobic zipper generated by surface-exposed non-polar residues on both faces. These include Phe169, Phe182, and Tyr222 (MreC); Leu69′, Phe71′, Tyr127′, and Tyr134′ (PBP2); Phe169 (MreC); and Tyr134′ (PBP2), forming a *π*–*π* stacking pair (Fig. 2b). Notably, in the crystal structure of unbound, closed PBP2, hydrophobic zipper residues Phe71′, Tyr127′, and Tyr134′ are covered by the anchor region (Fig. 3), indicating that binding to MreC requires uncovering of this apolar patch through the swaying movement. A structure-based sequence comparison of class B PBPs from rod-like bacteria using the closed form of *H. pylori* PBP2 indicates that residues that line the head/anchor interface display clear similarities among class B enzymes (Fig. 2c). In addition, in structures of unbound class B PBPs from both Gram-positive and Gram-negative bacteria, analogous hydrophobic patches are covered by their respective anchor regions (Fig. 3).

In order to further characterize the “opening” mechanism of the N-terminal region of PBP2, we generated a “disulfide locked” form of PBP2 by engineering two cysteine residues at sites Tyr134′ and Ala218′, predicted to be well-positioned to form a disulfide bond^19^. This mutant was unable to bind MreC in conditions where wild-type PBP2 could generate a stable complex in gel filtration (Supplementary Fig. 2). This indicates that PBP2 can only accommodate MreC when its anchor region is moved away from the head, and that the swaying mechanism is essential for binding. These data suggest that, in the context of elongasome formation, the role of the PBP anchor region could be to shield the head from untimely interaction with MreC and/or other protein partners, and that the N-terminal regions of PBPs play the role of protein interaction platforms, as previously hypothesized^3, 20, 21^.

### The PBP2:MreC interaction is essential in vitro and in vivo

In order to characterize the importance of the hydrophobic zipper for the PBP2:MreC interaction, we generated mutant forms of MreC encompassing triple mutations where Phe169, Phe182, and Tyr222 were all mutated to Ala (MreC-3A) or Asp (MreC-3D), as well as a double mutant where the two residues at the “edge” of the hydrophobic zipper, Arg154 and Thr221 (Fig. 2b), were mutated to Asp and Arg, respectively. While the interaction between PBP2 and wild-type MreC was characterized by a *K*_D_ of 0.4 μM by isothermal titration calorimetry (ITC), neither of the triple mutants were able to display any affinity for PBP2 either in gel filtration or ITC experiments (Supplementary Fig. 3). In order to exclude the possibility that this effect was due to poorly folded MreC-3A and MreC-3D variants, we performed circular dichroism (CD) experiments that showed that the two mutant forms display very similar folds to wild-type MreC (Supplementary Fig. 4). Notably, the MreC-R154D-T221R mutant was able to form a stable complex with PBP2 much like wild-type MreC, indicating that the three central residues in the PBP2:MreC interaction play the key stabilization role.

**Fig. 4:**
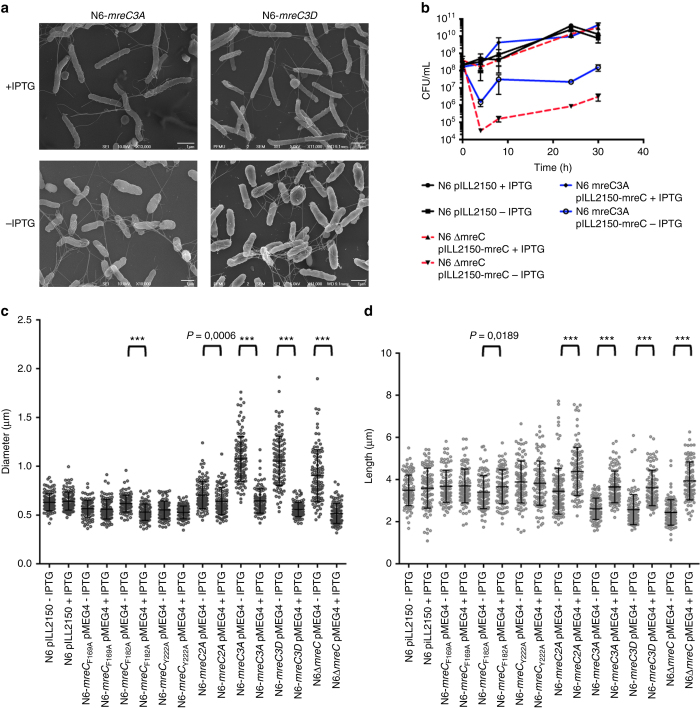
Determination of the effects of the 3A and 3D mutations on MreC’s hydrophobic zipper on *H. pylori*. **a** The *mreC3A* and *mreC3D* conditional mutants (N6 *mreC3A* pMEG4 and N6 *mreC3D* pMEG4) were grown in the presence or absence of IPTG (1 mM). Cell shape was monitored by scanning electron microscopy (representative images of bacteria after 24 h of growth are illustrated) and compared to the wild-type strain N6 pILL2150. **b** The control strain N6 pILL2150, the conditional *mreC* mutant (N6 ∆*mreC* pMEG4), and the 3A mutant (N6 *mreC3A* pMEG4) were grown in liquid culture in the presence or absence of IPTG. Viable bacteria were monitored by measuring the number of colony forming units (CFU/mL) as a function of time of growth (h); **c**, **d** Changes in cell shape in the presence or absence of IPTG (1 mM) were monitored by measuring bacterial cell diameter using the ImageJ software. Each *dot* represents the measured diameter (**c**) and length (**d**) of one individual bacterium. The difference in diameter (and length) in the presence or absence of IPTG was statistically significant for strains N6 ∆*mreC* pMEG4 and N6 *mreC3A* pMEG4 (unpaired two-tailed student *t*-test; ns = not significant; ***P < 0.0001 with a minimum of 100 bacteria being measured per strain and per growth condition). All other strains behaved similarly to the control strain N6 pILL2150

Having determined the importance of the hydrophobic zipper for the PBP2:MreC interaction in vitro and in order to verify the effect of the mutations in a cellular setting, we constructed conditional *H. pylori* mutants carrying individual point mutations within the hydrophobic patch of MreC, as well as double (2A) or triple mutations (3A or 3D), and studied the effect of IPTG depletion on cell growth. The strains carried the mutated *mreC* genes in the *mreC* native locus, and a wild-type copy of *mreC* on plasmid pMEG4 under control of an IPTG inducible promoter. Under permissive conditions, all strains grew normally as the control strain N6 pILL2150. However, in the absence of IPTG, strains carrying the triple (but not single or double) mutations stopped growing and cells became enlarged (Fig. 4a, b, and Supplementary Fig. 5) as evidenced by the increase in cell diameter and decrease in cell length (Fig. 4c, d). These results indicate that in the absence of wild-type MreC, neither MreC-3A nor MreC-3D are able to sustain growth and cell shape of *H. pylori*, underlining the importance of an intact PBP2:MreC hydrophobic zipper for bacterial growth and shape. Interestingly, although single and double mutations did not impair growth, the single mutation F182A and the double mutant F169A-F182A presented altered shape dimensions. In the absence of IPTG, the two mutants presented an increased diameter and a reduced length. These differences were exacerbated in the double mutant, particularly the decrease in cell length (Fig. 4c, d and Supplementary Fig. 5). Hence, the accumulation of mutations in the hydrophobic zipper has a gradual impact on the functionality of the PBP2:MreC complex.

### MreC as a scaffold for elongasome formation

MreC has been shown to form helical or patch-like patterns on membranes of a variety of rod-like cells, and interact with itself on a bacterial two-hybrid assay^7, 9, 11, 12, 17^. In addition, crystal structures of MreC from different species^17, 18^, including the work presented here, indicate that all MreC variants studied to date have the tendency to self-associate (Supplementary Fig. 6). The fact that MreC patches are yet to be characterized in vitro suggests that MreC’s self-association may be weak or transient, requiring the stability of a crystalline environment for visualization. However, in a cellular context, this association could be facilitated by the high abundance of MreC (12,000 copies/cell)^17^, leading to banded patterns observed.

## Discussion

**Fig. 5:**
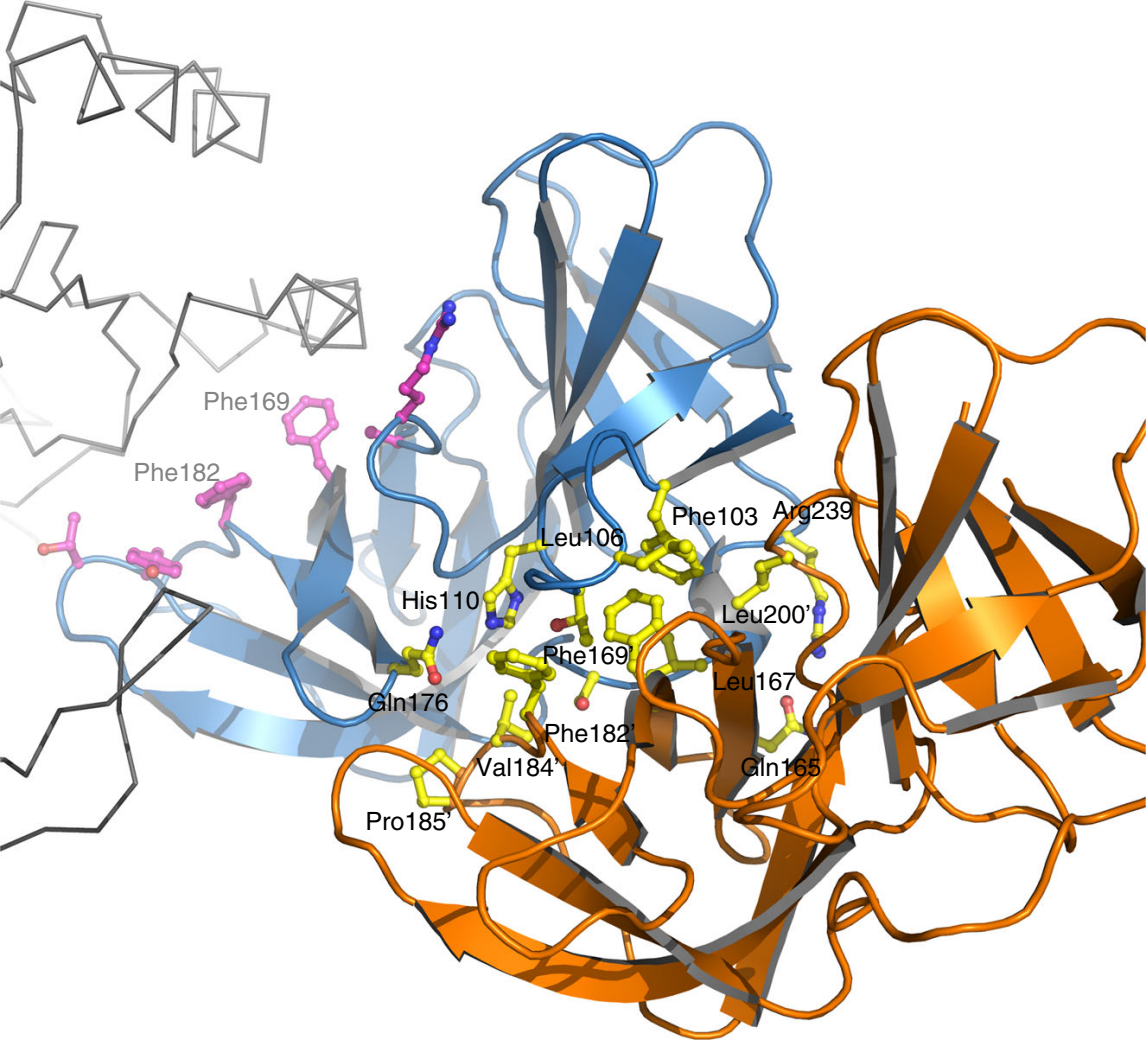
MreC employs different surfaces in order to maximize interactions with partner molecules. MreC (*blue*) interacts both with PBP2 (*gray ribbon*) and with another MreC butterfly (*orange*). The latter recognition region is composed mostly of hydrophobic interactions, with few exceptions (*yellow sticks*). Residues Phe169 and Phe182, localized at the center of the MreC–MreC interaction platform that is provided by a neighboring molecule (with *apostrophes*), are also involved in the formation of the hydrophobic zipper that interacts with PBP2 (*purple sticks*). This suggests that MreC can modify the protein partner with which it interacts through a slight adjustment of the residues available for binding

MreC has been suggested to play a structural role in cell wall synthesis. MreC variants from different species co-localize with high molecular weight PBPs, lytic transglycosylases, and outer membrane proteins, but also with MreB polymers. These observations have led to the implication of MreC as a scaffold that stabilizes elongasome-forming proteins^7–9, 11, 12, 17, 22, 23^. However, how a 28 kDa molecule can serve as a scaffold that permits the association of a variety of elongasome partners has remained obscure. Our data shed light on this question by revealing that MreC employs two distinct surfaces for partner recognition. The first region of the MreC “butterfly” interacts with the N-terminus of PBP2, as described above, while a second one, located at the junction of the two butterfly “wings”, tethers at the center of partner MreC molecule (Fig. 5), involving a total of 1800 Å^2^ of total buried area (calculated with PISA, Table 1; ref. ^24^). In the cell, the absolute position of MreC and PBP2/elongasome partners on the membrane could be dependent on the flexibility of their extreme N-termini that connect to their transmembrane helices; despite the fact that the TM helices of PBP2 and MreC are missing from our structures, both N-termini point in the same direction, suggesting that they could employ a proximal membrane attachment site to favor interaction. In addition, the inherent flexibility of the N-terminus of PBP2 could allow it to adjust its position in order to interact with other cell wall-synthesis molecules (such as class A PBPs and membrane-embedded RodA^17, 25^) while still being scaffolded by MreC, which itself can bind to carboxypeptidases, endopeptidases, and lytic transglycosylases^8^.

**Table 1:** Pisa assembly list

Molecule composition	Surface area (Å^2^)	Buried area (Å^2^)	ΔG^int^ (kcal/mol)
PBP2:MreC1:MreC2 Complex 1	38,960	4060	−29.4
PBP2:MreC1:MreC2 Complex 2	39,720	3500	−27.3
PBP2:MreC1 Complex 1	32,100	2020	−14.4
PBP2:MreC1 Complex 2	32,560	1770	−14.7
MreC1:MreC2 Complex 1	15,687	1806	−13.2
MreC1:MreC2 Complex 2	15,889	1726	−12.6

In the model organism *E. coli*, cell wall complexes can be stably detected during the exponential phase of cell growth, but not during stationary phase^14^. This indicates that proteins need to be able to rapidly associate and dissociate at specific moments of the cell cycle; these data are supported by observations in other organisms such as *C. crescentus*, where PBP2 was shown to associate to the cell wall synthesis machinery dynamically and transiently^10^. The fact that hydrophobic interactions (more feeble than polar interactions) are involved in PBP2:MreC recognition indicates that these complexes can form and be disrupted as needed during the cell wall elongation process. The key role played by PBP2 and MreC in cell wall elongation in all bacteria studied to date indicates that our PBP2:MreC complex structure likely represents a snapshot of a step in elongasome formation that is common to all non-spherical bacteria.

## Methods

### Protein purification

Proteins were purified and the complex was formed as previously described^8^. Briefly, for PBP2, BL21 (DE3) Star cells (Novagen) carrying plasmid pACYDuet-PBP2 (res L38-L588) were grown at 37 °C in LB liquid medium supplemented with chloramphenicol (30 mg mL^−1^). When the absorbance at 600 nm reached 0.6 AU, protein expression was induced by the addition of 1 mM IPTG. Growth was continued overnight at 18 °C. The cell pellet was resuspended in buffer A (50 mM Tris-HCl pH 8.0, 300 mM NaCl, 0.25% CHAPS (3-[(3-Cholamidopropyl-dimethylammonio]-1-propanesulfonate (SIGMA)), 5% glycerol) and the cells were disrupted by using a cell disruptor (Constant Systems). After centrifugation, the soluble fraction containing the His_6_-tagged PBP2 was purified over a nickel-nitrilotriacetate (Ni^2+^-NTA) column and the protein was eluted with an imidazole gradient.

GST-MreC, which harbors a thrombin cleavage sequence between GST and MreC (residues L37-N248), was purified by employing a similar protocol, with the exception that BL21 (DE3) Star cells (Novagen) were employed. The pellet was resuspended in buffer A (to which 1 mM EDTA was added) and cells were disrupted using a cell disruptor (Constant Systems). The supernatant was loaded onto a GST-column (GE Healthcare) and eluted in buffer B (buffer A + 20 mM glutathione).

His_6_-PBP2 and GST-MreC were incubated in a 1.2:1 ratio and injected onto a Superdex HR10/30 column (GE Healthcare) that had been previously equilibrated in buffer C (25 mM Tris-HCl pH 8.5, 100 mM NaCl, 1 mM EDTA). The complex peak was isolated and loaded onto a GST column in the same buffer. In order to remove the GST tag, 0.01 units of thrombin were added per microgram of loaded complex to the column overnight, after which the GST-free complex was eluted. The sample was concentrated for crystallization trials.

### Crystallization of PBP2 and PBP2:MreC

PBP2 and PBP2:MreC complex crystals were grown by the vapor diffusion method at 20 °C using a hanging-drop setup. PBP2 was crystallized by mixing 2 μL of protein sample (1.4 mg mL^−1^, 25 mM HEPES pH 7.5, 100 mM NaCl, 1 mM EDTA) and 2 μL of reservoir solution (1.4 M (NH_4_)_2_SO_4_, 50 mM HEPES-Na pH 7.0). Crystals of the PBP2:MreC complex were grown using a protein sample at 6 mg mL^−1^ in 25 mM Tris-HCl pH 8.0, 100 mM NaCl, 1 mM EDTA and by mixing with a reservoir solution containing 5% w/v PEG 6000, 50 mM Citric acid pH 5.0, 9 mM ZnCl_2_. PBP2 and PBP2:MreC crystals were cryoprotected with Paratone and 20% glycerol, respectively, mounted on cryoloops and flash-cooled under liquid nitrogen. X-ray diffraction data were collected under a cold nitrogen stream at 100 K at the European Synchrotron Radiation Facility (ESRF, Grenoble, France). The experimental set up of the beamline and data quality of the collected images were monitored with MxCuBE^26, 27^ and ADXV (http://www.scripps.edu/~arvai/adxv.html).

### Structure determination and refinement

X-ray diffraction images for both structures were indexed and scaled with XDS^28^. XDSGUI (http://strucbio.biologie.uni-konstanz.de/xdswiki/index.php/XDSGUI) was used to perform a checkup of data quality and resolution cutoff^29–33^. The reduced X-ray diffraction data was imported in to the CCP4 program suite^34^. The PBP2 structure was solved by molecular replacement using the structure of PBP3 from *P. aeruginosa* ^15^ as a model and employing BALBES^35^. Generation of the initial PBP2 model was performed following spatial sampling methods using ROSETTA as implemented in PHENIX^36^ to improve the convergence radius^37^. Cycles of manual building, phase optimization, and automatic model-building were extensively performed with COOT^38^, PARROT^39^, and BUCCANNER^40^. Refinement cycles, including the local-NCS option, were performed using REFMAC^41^. After several cycles of manual model building and refinement, the PBP2 model was good enough to allow the generation of a molecular replacement solution for the PBP2:MreC complex data set using PHASER^42^. This initial model, containing just two molecules of PBP2 in the ASU, was submitted to the same procedure of model building and refinement mentioned above; at later steps, four molecules of MreC were built per ASU. Once manual model building and refinement converged^43^, the TLS option^44, 45^ was introduced in REFMAC. The stereochemical quality of the refined models was verified with MOLPROBITY^46^, as implemented in COOT and PROCHECK^47^. The secondary structure assignment was performed by DSSP^48^ and STRIDE^49^. X-ray diffraction data, structure solution, and refinement statistics are found in Table 2. Figures displaying protein structures were generated with PyMol 1.7 (http://www.pymol.org).

**Table 2:** Data collection, molecular replacement, and structure refinement statistics

Data set	PBP2	PBP2:MreC
*Data collection*		
X-ray source	BM30A	ID23EH2
Detector	ADSC Q315R	MARCCD 225
Wavelength (Å)	0.979526	0.87260
Scan-range (^o^)	182	199
Oscillation (^o^)	1	1
Space group	P2_1_	C2
*a* (Å)	71.40	338.66
*b* (Å)	140.96	48.34
*c* (Å)	81.30	151.51
β (°)	101.7	113.01
Overall resolution (Å)	45.39–3.03	43.94–2.74
No. observed/unique reflections	81345/27837	144817/52464
High-resolution shell (Å)	3.21–3.03	2.90–2.74
Completeness (%) (last shell)	90.8 (84.5)	86.5 (83.1)
*R*_sym_ (last shell)	12.0 (43.4)	7.3 (41.0)
*I/σ(I)* (last shell)	12.15 (3.0)	19.84 (3.0)
CC_1/2_	98.9 (85.4)	99.7 (89.9)
Wilson plot B-factor (Å^2^)	37.46	45.79
*Molecular replacement*		
Mol/ASU	2(PBP2)	2(PBP2), 4(MreC)
Balbes probability (%) (2(PBP2))	89.72	—
Phaser LLG score (2(PBP2))	—	487
*Refinement*		
Initial *R*_work_/*R*_free_ (%)	43.37/46.74	47.74/50.77
Final *R*_work_/*R*_free_ (%)	25.10/28.77	25.69/29.22
RMS deviation, bond lengths (Å)	0.010	0.008
RMS deviation, bond angles (°)	1.268	1.269
Mean B-factor (Å^2^)	51.47	60.03
No. of protein atoms	8332	13312
No. of water molecules	24	69
No. of sulfate molecules	3	
Residues in most favored/allowed region of Ramachandran plot (%)	99.8	99.8

### Generation of MreC hydrophobic zipper and PBP2 Cys mutants

Primers for MreC mutants were designed using the NEBase Changer^TM^ tool (New England Biolabs, http://nebasechanger.neb.com). For each of the mutated residues in the triple mutants (Phe169, Phe182, and Tyr222), four primers were designed: one reverse and three forward, each of them harboring a single point mutation to either Alanine or Aspartic acid. Prior to amplification (with PhusionR High Fidelity DNA Polymerase, NEB), the primers were phosphorylated using PNK (Fermentas/Thermo). The pGEX4T-MreC construct was used as a template for the mutagenesis reaction. The resulting linearized and 5′-phosphorylated blunt version of the final construct was then treated with DpnI to digest the template plasmid, purified on an agarose gel and used alone in a ligation reaction in order to generate the final construct. Mutations were performed sequentially, i.e., three rounds for the triple mutants (3A and 3D) and two rounds for the double mutant (R154D-T221R). Each round of mutagenesis was confirmed by sequencing.

In order to select sites for introduction of Cys mutations within the PBP2 sequence, we employed Disulfide by Design 2 server (http://cptweb.cpt.wayne.edu/DbD2/), which identified Tyr134′ and Ala218′ as optimal candidate sites. For introduction of mutations, the pACYC-Duet-PBP2 vector was used as a template. Two pairs of primers were designed using the same strategy as described above for MreC mutations.

### Size-exclusion chromatography assays

Gel filtration assays were performed a Superdex HR10/30 column (GE Healthcare) that had been previously equilibrated in 25 mM Tris-HCl pH 8.5, 100 mM NaCl, 1 mM EDTA. PBP2 was incubated with MreC (wild type), MreC-3A, MreC-3D, or MreC-R154D-T221R for 2 h prior to injection into the column. 0.5 mL fractions were collected for all experiments. Experiments performed with PBP2-Y134C-A218C used the same buffer and parameters as described above, with the exception of the gel filtration assay in the presence of TCEP, where 1 mM of the reducing agent was added. All MreC forms carry the GST tag.

### Isothermal titration calorimetry

ITC experiments were carried out with iTC200 microcalorimeter (MicroCal, Northampton, MA) at 20 °C. A stirring speed of 300 rpm and a reference power of 5 μcal/s were used. In each measurement, PBP2 (3 μL of 160.4 μM) were titrated into the sample cell containing MreC (wild type, 3A, or 3D variants at 25 μM, GST tagged). The reference cell was filled with water. Raw ITC data were evaluated using the Origin software (Northampton, MA).

### Circular dichroism

CD spectra were recorded between 195 and 260 nm on a J-810 Jasco spectropolarimeter using a quartz cuvette with a path length of 0.5 mm, a 100 nm/min scanning speed, and a bandwidth of 1 nm. Each variant (wild-type MreC, MreC-3A, and MreC-3D) was tested in PBS in different concentrations (4.5, 11.0, and 20 µM). Twenty spectra were measured at room temperature, averaged and corrected for buffer contribution. Experimental data were analyzed using the BeStSel server (http://bestsel.elte.hu).

### Construction of the *H. pylori mreC* mutants

The *mreC* locus was cloned into pUC18-Gm as follows: 500-bp of the *mreB* gene upstream of *mreC* and the *mreC* gene were amplified by PCR using primers 1373-F (CTAAAAGCATTAGAGTGGCTGGGG) and 1372-R-Kpn1 (CGCGGTACCCCGCTAGTTTTTCACATCGCTCAAAAACAC) and cloned into the pUC18-Gm plasmid^50^ upstream of the non-polar gentamicin cassette. The 500-bp region downstream of *mreC* was amplified using primers 1371-BamH1 (CGCGGATCCGCGACTAGCCTTATATTCAAGGCTAATAAGCG) and 1371-R-Xba1 (GCTCTAGAGCGCATGTGGTTAAAGTAGAAAGCGAGAG) and cloned into the pUC18-Gm downstream of the non-polar cassette generating the suicide pUC18-*mreC*-Gm plasmid. The suicide plasmid was used as a template to introduce mutations into the *mreC* gene by inverse PCR. The different variants of mutated *mreC* were introduced into *H. pylori* by natural transformation^51^ using the *mreC* conditional strain N6∆*mreC* pMEG4 as a recipient strain^8^. This strain has the chromosomal copy of *mreC* replaced by a non-polar kanamycin cassette and a wild-type *mreC* gene on the pMEG4 plasmid under control of an IPTG inducible promoter. By transforming plasmid pUC18-*mreC*-Gm carrying each of the different mutations into strain N6*∆mreC* pMEG4 and selecting for gentamycin resistance (5 µg/mL) and growth with 1 mM of IPTG, we selected the allelic exchange of the kanamycin resistance cassette by the *mreC*-Gm construction. Isolated clones were confirmed to be gentamycin resistant and kanamycin sensitive. The mutated chromosomal *mreC* locus was confirmed by sequencing. As an example, we obtained strain N6-*mreC*3A pMEG4 carrying the mutated *mreC*3A in the original *mreBC* locus and a wild-type copy of *mreC* on plasmid pMEG4 under control of IPTG. *H. pylori* was cultivated microaerobically (Anoxomat; final concentration of N_2_:CO_2_:0_2_ of 84:10:6%) at 37 °C on blood agar or on liquid medium consisting of brain–heart infusion (Oxoid) with 10% fetal calf serum (Eurobio) supplemented with an antibiotic-antifungal mix^52^. The mutant strains were grown with or without IPTG at 1 mM. Bacteria were sampled during the growth curve to estimate cell viability (CFU/mL). Samples were also collected at 24 h of growth and analyzed by microscopy using an Axio Observer (Zeiss) instrument. The diameter and length of individual bacteria was measured using ImageJ software. The same samples were prepared for scanning electron microscopy as previously described^8^.

### Data availability

Coordinates and structure factors for PBP2 and PBP2:MreC have been in the Protein Data Bank with access codes 5LP4 and 5LP5. The data that support the findings of this study are available from the corresponding author upon request.

## Electronic supplementary material

Supplementary Information

## References

[1] Silver LL (2013). Viable screening targets related to the bacterial cell wall. Ann. N. Y. Acad. Sci..

[2] den Blaauwen T, de Pedro MA, Nguyen-Distèche M, Ayala JA (2008). Morphogenesis of rod-shaped sacculi. FEMS Microbiol. Rev..

[3] Höltje JV (1998). Growth of the stress-bearing and shape-maintaining murein sacculus of *Escherichia coli*. Microbiol. Mol. Biol. Rev..

[4] Matteï P-J, Neves D, Dessen A (2010). Bridging cell wall biosynthesis and bacterial morphogenesis. Curr. Opin. Struct. Biol..

[5] Kruse T, Bork-Jensen J, Gerdes K (2005). The morphogenetic MreBCD proteins of *Escherichia coli* form an essential membrane-bound complex. Mol. Microbiol..

[6] Figge RM, Divakaruni AV, Gober JW (2004). MreB, the cell shape-determining bacterial actin homologue, coordinates cell wall morphogenesis in *Caulobacter crescentus*. Mol. Microbiol..

[7] Dye NA, Pincus Z, Theriot JA, Shapiro L, Gitai Z (2005). Two independent spiral structures control cell shape in *Caulobacter*. Proc. Natl. Acad. Sci. USA.

[8] El Ghachi M (2011). Characterization of the elongasome core PBP2:MreC complex of *Helicobacter pylori*. Mol. Microbiol..

[9] Leaver M, Errington J (2005). Roles for MreC and MreD proteins in helical growth of the cylindrical cell wall in *Bacillus subtilis*. Mol. Microbiol..

[10] Lee TK (2014). A dynamically assembled cell wall synthesis machinery buffers cell growth. Proc. Natl. Acad. Sci. USA.

[11] Divakaruni AV, Baida C, White CL, Gober JW (2007). The cell shape proteins MreB and MreC control cell morphogenesis by positioning cell wall synthetic complexes. Mol. Microbiol..

[12] Divakaruni AV, Ogorzalek Loo RR, Xie Y, Loo JA, Gober JW (2005). The cell-shape protein MreC interacts with extracytoplasmic proteins including cell wall assembly complexes in *Caulobacter crescentus*. Proc. Natl. Acad. Sci. USA.

[13] Vats P, Shigh Y-L, Rothfield L (2009). Assembly of the MreB-associated cytoskeletal ring of *Escherichia coli*. Mol. Microbiol..

[14] Trip E, Scheffers D-J (2015). A 1MDa protein complex containing critical components of the *Escherichia coli* divisome. Sci. Rep..

[15] Han S (2010). Structural basis for effectiveness of siderophore-conjugated monocarbams against clinically relevant strains of *Pseudomonas aeruginosa*. Proc. Natl. Acad. Sci. USA.

[16] Liu Y, Möller MC, Petersen L, Söderberg CAG, Hederstedt L (2010). Penicillin-binding protein SpoVD disulphide is a target for StoA in *Bacillus subtilis* forespores. Mol. Microbiol..

[17] van den Ent F (2006). Dimeric structure of the cell shape protein MreC and its functional implications. Mol. Microbiol..

[18] Lovering AL, Strynadka NC (2007). High resolution structure of the major periplasmic domain from the cell shape-determining filament MreC. J. Mol. Biol..

[19] Craig DB, Dombkowski AA (2013). Disulfide by design 2.0: a web-based tool for disulfide engineering in proteins. BMC Bioinformatics.

[20] Macheboeuf P, Contreras-Martel C, Job V, Dideberg O, Dessen A (2006). Penicillin binding proteins: key players in bacterial cell cycle and drug resistance processes. FEMS Microbiol. Rev..

[21] Powell AJ, Tomberg J, Deacon AM, Nicholas RA, Davies C (2009). Crystal structures of penicillin-binding protein 2 from penicillin-susceptible and -resistant strains of *N. gonorrhoeae* reveal an unexpectedly subtle mechanism for antibiotic resistance. J. Biol. Chem..

[22] White CL, Kitich A, Gober JW (2010). Positioning cell wall synthetic complexes by the bacterial morphogenetic proteins MreB and MreD. Mol. Microbiol..

[23] Slovak PM, Porter SL, Armitage JP (2006). Differential localization of Mre proteins with PBP2 in *Rhodobacter sphaeroides*. J. Bacteriol..

[24] Krissinel E, Henrick K (2007). Inference of macromolecular assemblies from crystalline state. J. Mol. Biol..

[25] Banzhaf M (2012). Cooperativity of peptidoglycan synthases active in bacterial cell elongation. Mol. Microbiol..

[26] Leal RM (2011). Experimental procedure for the characterization of radiation damage in macromolecular crystals. J. Synchrotron Radiat..

[27] Gabadinho J (2010). MxCuBE: a synchrotron beamline control environment customized for macromolecular crystallography experiments. J. Synchrotron Radiat..

[28] Kabsch W (2010). XDS. Acta Cryst. D.

[29] Evans PR, Murshudov GN (2013). How good are my data and what is the resolution?. Acta Cryst. D.

[30] Karplus PA, K D (2012). Linking crystallographic model and data quality. Science.

[31] Diederichs K, PA K (1997). Improved R-factors for diffraction data analysis in macromolecular crystallography. Nat. Struct. Biol..

[32] Diederichs K, P.A. K (2013). Better models by discarding data?. . Acta Cryst. D.

[33] Karplus PA, Diederichs K (2015). Assessing and maximizing data quality in macromolecular crystallography. Curr. Opin. Struct. Biol..

[34] Winn MD (2011). Overview of the CCP4 suite and current developments. Acta Cryst. D.

[35] Long F, Vagin AA, Young P, Murshudov GN (2008). BALBES: a molecular-replacement pipeline. Acta Cryst. D.

[36] Adams PD (2010). PHENIX: a comprehensive python-based system for macromolecular structure solution. Acta Cryst. D.

[37] DiMaio F (2013). Improved low-resolution crystallographic refinement with Phenix and Rosetta. Nat. Methods.

[38] Emsley P, Cowtan K (2004). Coot: model-building tools for molecular graphics. Acta Cryst. D.

[39] Cowtan K (2010). Recent developments in classical density modification. Acta Cryst. D.

[40] Cowtan K (2006). The Buccaneer software for automated model building. 1. Tracing protein chains. Acta Cryst. D.

[41] Murshudov GN (2011). REFMAC5 for the refinement of macromolecular crystal structures. Acta Cryst. D.

[42] McCoy AJ (2007). Phaser crystallographic software. J. Appl. Crystallogr..

[43] Brünger AT (1997). Free R value: cross-validation in crystallography. Methods Enzymol..

[44] Painter J, Merritt EA (2006). Optimal description of a protein structure in terms of multiple groups undergoing TLS motion. Acta Cryst. D.

[45] Painter J, Merrit EA (2006). TLSMD web server for the generation of multi-group TLS models. J. Appl. Crystallogr..

[46] Chen VB (2010). MolProbity: all-atom structure validation for macromolecular crystallography. Acta Cryst. D.

[47] Laskowski RA, MacArthur MW, Moss DS, Thornton JM (1993). PROCHECK: a program to check the stereo chemical quality of protein structures. J. Appl. Crystallogr..

[48] Kabsch W, Sander C (1983). Dictionary of protein secondary structure: pattern recognition of hydrogen-bonded and geometrical features. Biopolymers.

[49] Heinig M, Frishman D (2004). STRIDE: a web server for secondary structure assignment from known atomic coordinates of proteins. Nucleic Acids Res..

[50] Bury-Moné S (2003). Presence of active aliphatic amidases in Helicobacter species able to colonize the stomach. Infect. Immun..

[51] Skouloubris S, Thiberge JM, Labigne A, De Reuse H (1998). The *Helicobacter pylori* UreI protein is not involved in urease activity but is essential for bacterial survival in vivo. Infect. Immun..

[52] Bury-Moné S (2004). Responsiveness to acidity via metal ion regulators mediates virulence in the gastric pathogen *Helicobacter pylori*. Mol. Microbiol..

[53] Robert X, Gouet P (2014). Deciphering key features in protein structures with the new ENDscript server. Nucleic Acids Res..

