# Supplementary material for: Molecular architecture of the PBP2–MreC core bacterial cell wall synthesis complex
Source: Nat Commun. 2017 Oct 3;8:776. doi: 10.1038/s41467-017-00783-2 (PMC5626683; doi:10.1038/s41467-017-00783-2)
Supplement: Supplementary file 1 — Supplementary Information [file 41467_2017_783_MOESM1_ESM.pdf]

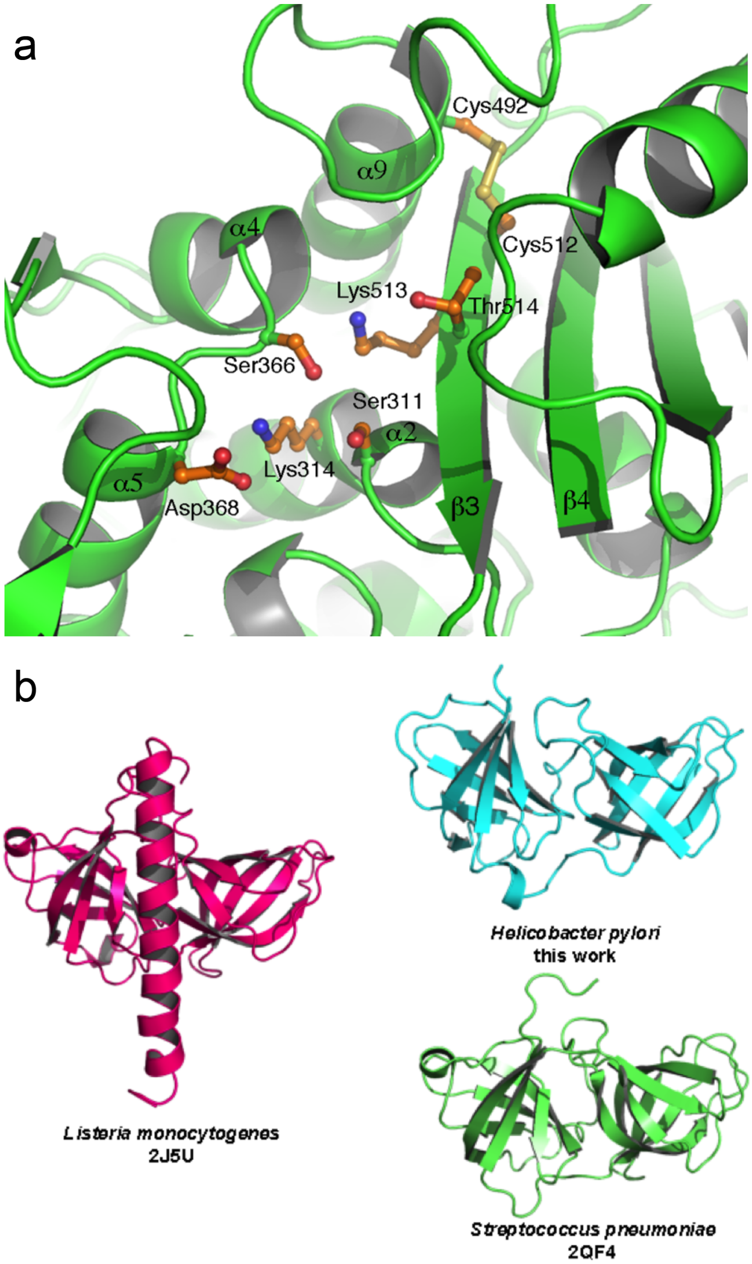

Supplementary Figure 1. (a) The active site of the transpeptidation domain of PBP2 harbors the classical fold of the 3 catalytic motifs: S-X-X-K (Ser311-Val312-Val313-Lys314), which includes the catalytic serine; S-X-N/D (Ser366-Val367-Asp368); and K-T/S-G (Lys513-Thr514-Gly515). A disulfide bond (Cys492-Cys512), unusual for PBPs, links β3 and α9. (b) Structures of monomers of MreC variants solved to date are highly reminiscent, displaying a 'two-winged butterfly' fold for both Gram-positive and Gram-negative organisms. Note that 2J5U also displays an additional elongated helix, located N-terminally to the butterfly fold, which despite being present in the clones of MreC from *H. pylori* (this work) and *S. pneumoniae* (2QF4) is not visible in either of the two structures.

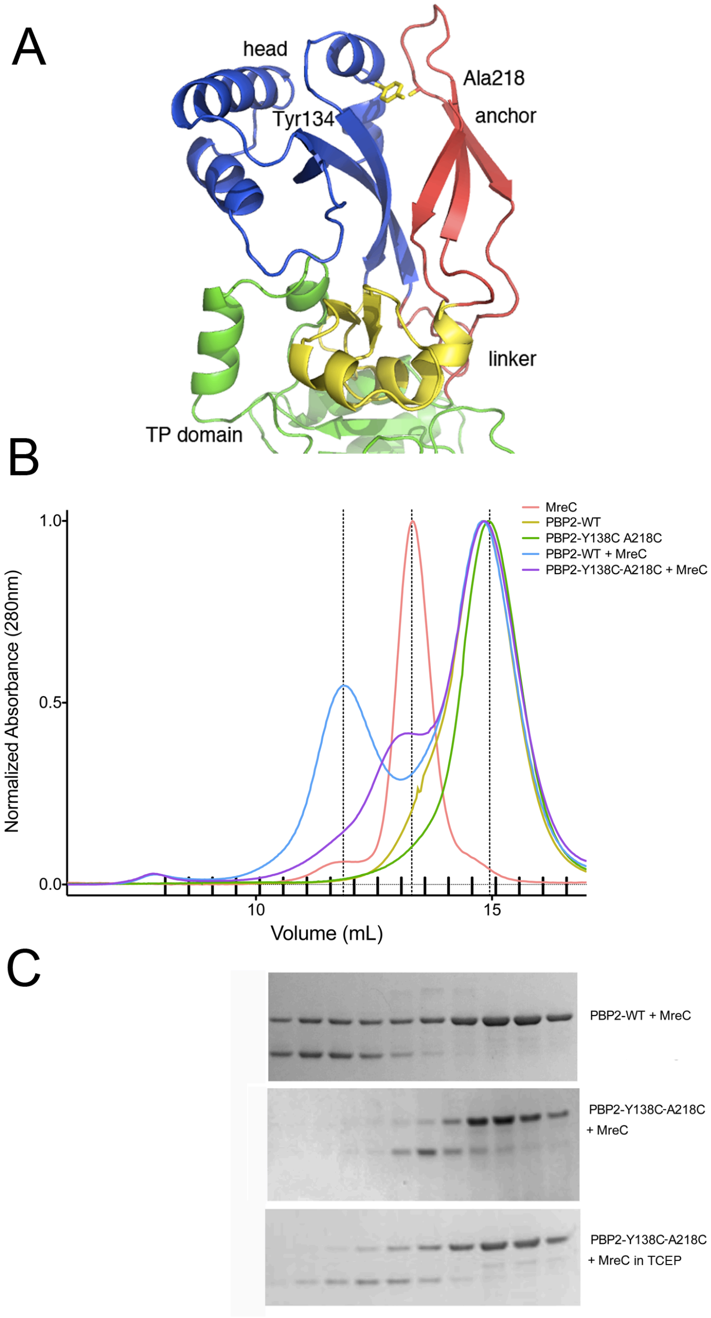

Supplementary Figure 2: The opening of the head and anchor regions is important for partner recognition. (A) Tyr134' and Ala218' were identified as optimal residues for mutation into cysteines for disulfide bond generation through employment of the Disulfide by Design server (http://cptweb.cpt.wayne.edu/DbD2/). (B) Gel filtration profiles of wild type and Cys-locked PBP2 in the presence of MreC. The complex generated between wild type PBP2 and MreC is not formed if Cys-locked PBP2 is employed; however, it can be partially recovered if gel filtration is performed in the presence of reducing agent TCEP (C). All MreC forms carry the GST tag.

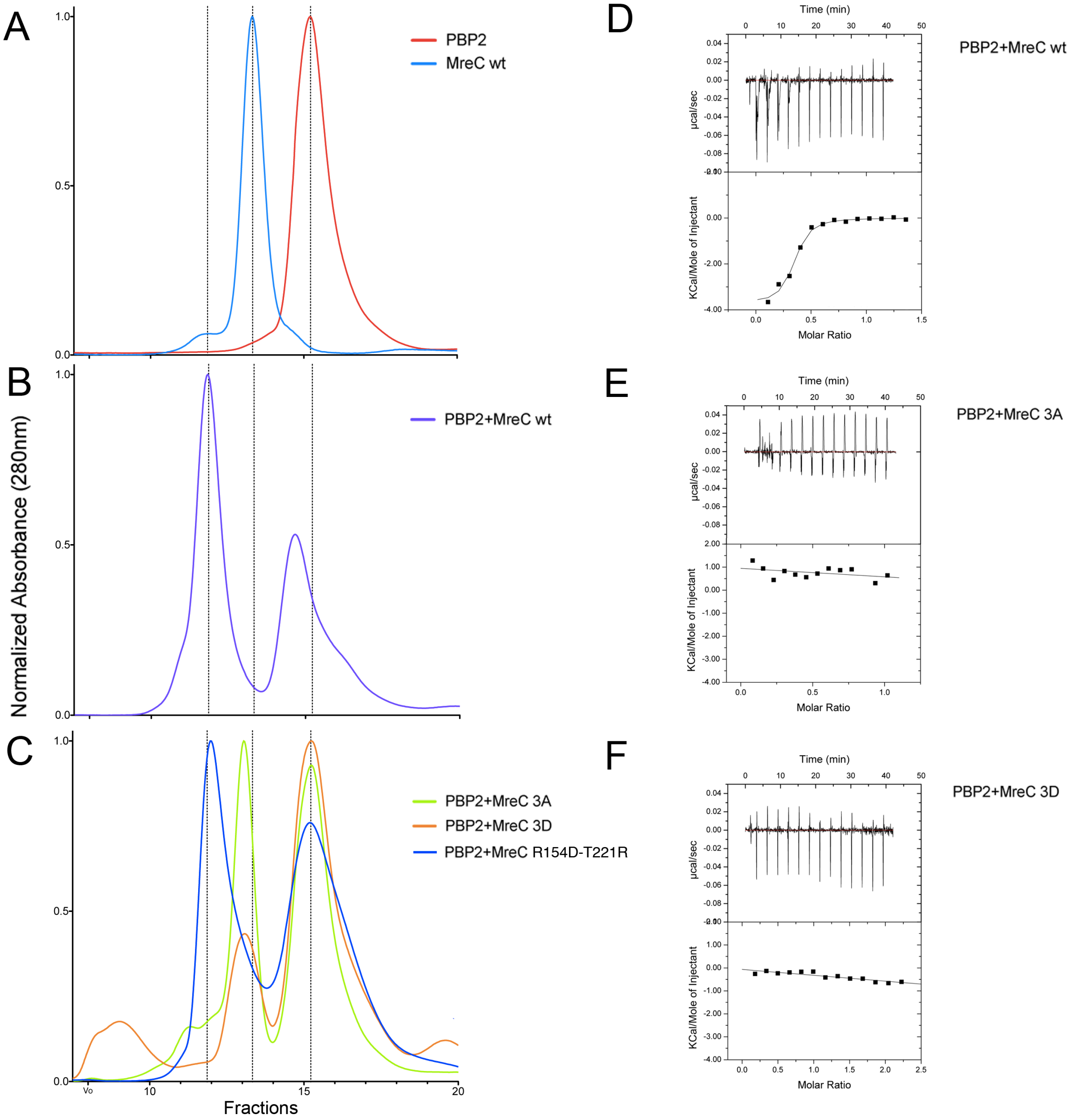

Supplementary Figure 3: The hydrophobic zipper of MreC is essential for the formation of a stable complex with PBP2. (A) PBP2 (red) and WT MreC (blue) bind stably to generate a complex that shifts to an earlier elution volume (left peak in B) in a Superdex200 column (GE Healthcare). (C) MreC mutants 3A and 3D, when incubated with PBP2 (yellow and orange lines), are unable to generate the same shift on gel filtration as seen for the wild type form, while the double MreC-R154D-T221R mutant behaves as wild type MreC (blue line). (D) Isothermal Titration Microcalorimetry (ITC) experiments indicate that wild type MreC binds to PBP2 with a Kd of 0.4 µM, while MreC 3A and 3D variants (E, F) show no affinity for PBP2. In all experiments, MreC was expressed as a GST fusion protein, as described in Materials & Methods.

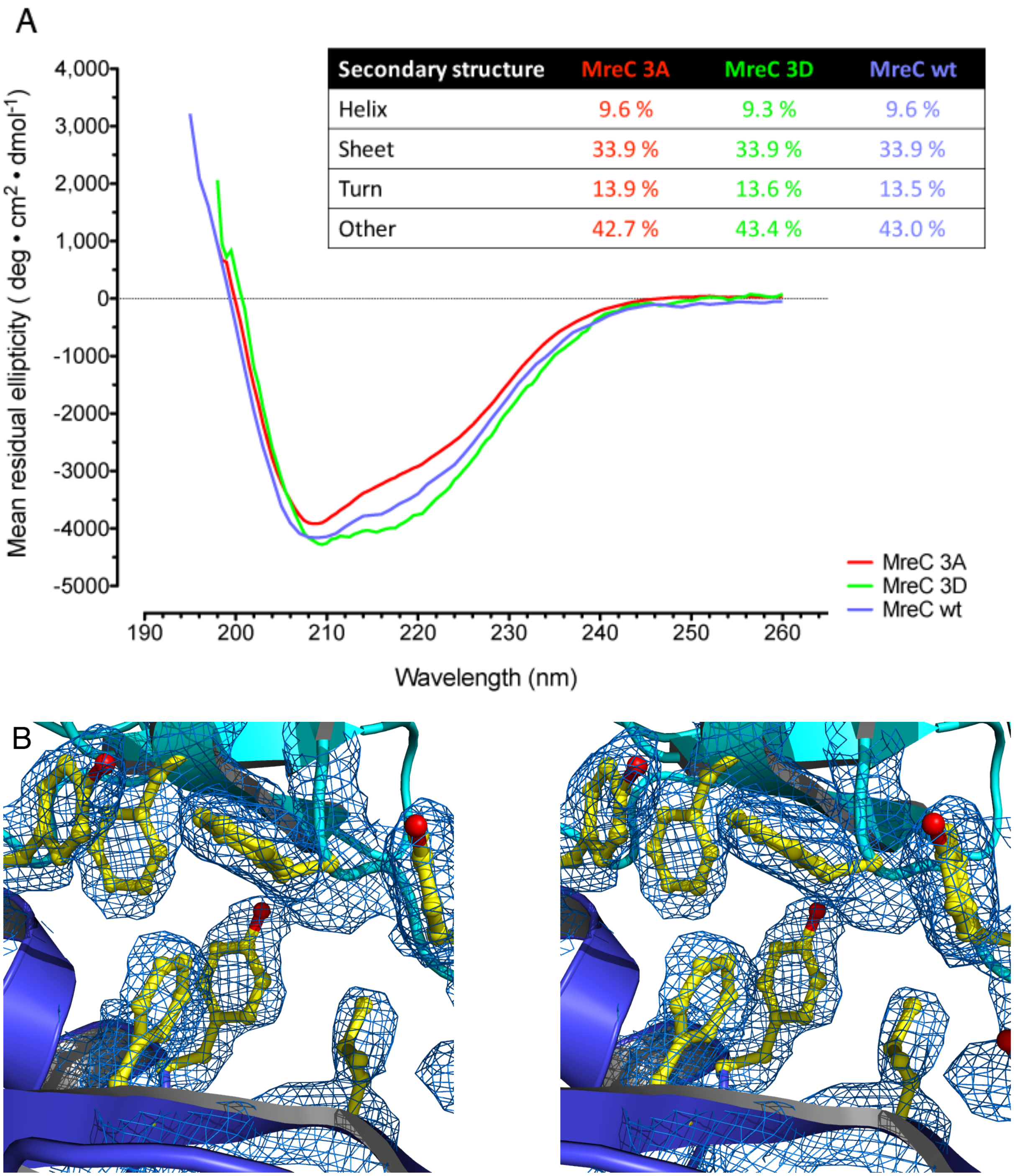

Supplementary Figure 4: The hydrophobic zipper of MreC does not disrupt MreC's fold. (A) Circular dichroism measurements of wild type MreC as well as MreC-3A and MreC-3D indicate that the overall folds of all three forms are comparable. These experiments were performed with GST-free MreC variants. (B) 2Fo-Fc map of the hydrophobic zipper region of the PBP2:MreC complex, calculated at 1.1 σ.

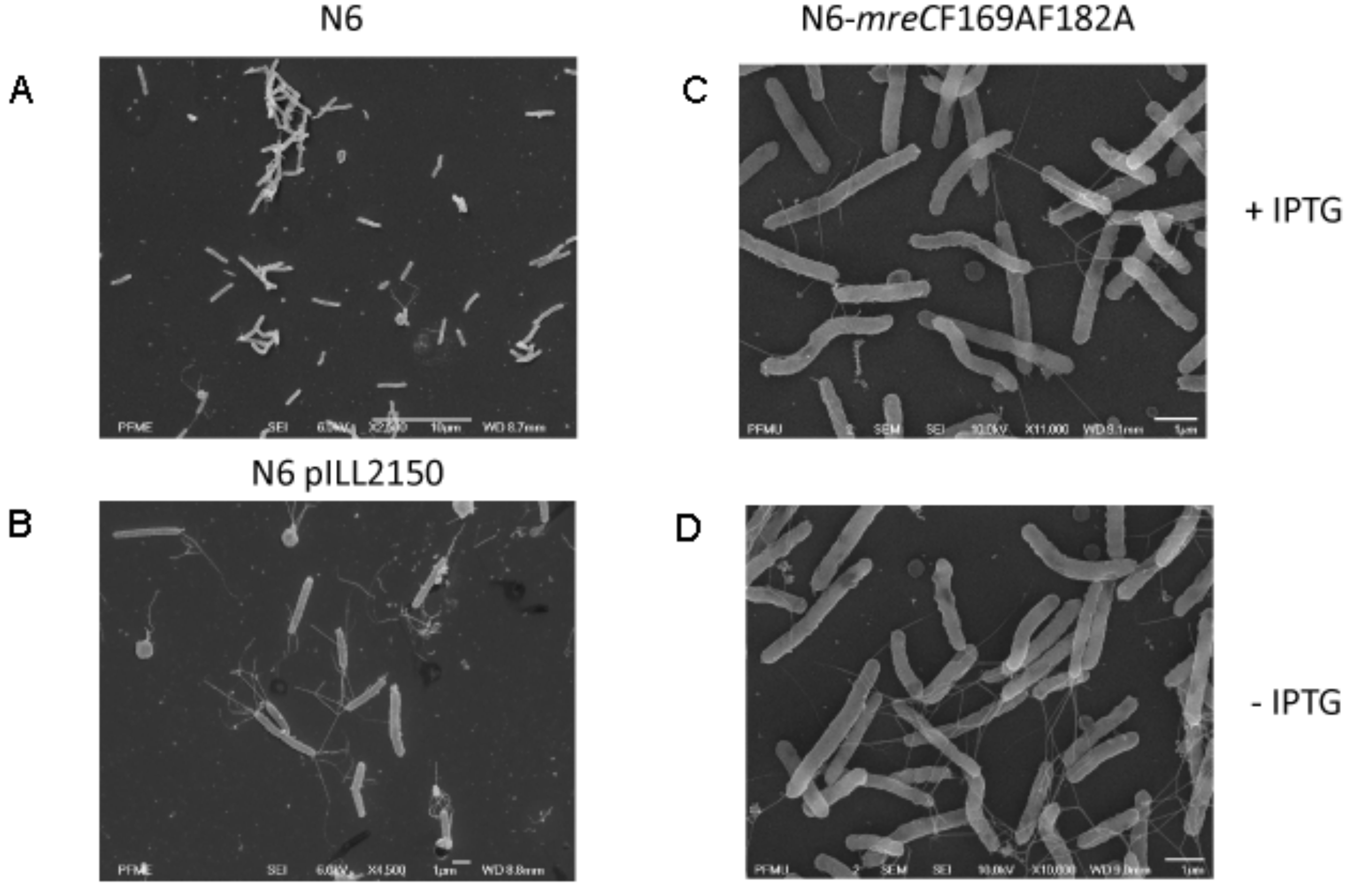

Supplementary Figure 5: The double mutant N6 *mreC2A* pMEG4, in which F169 and F182 were substituted by alanine, was constructed as an intermediate step to obtain the triple A mutant N6 mre*C3A* pMEG4. We tested whether a double mutation in the PBP2-MreC interphase was sufficient to interfere with complex formation and lead to loss of cell shape. The strain was grown with (A) or without (B) IPTG (1 mM). The double mutant was able to grow similarly to the control strain N6 pILL2150 even in the absence of IPTG (C, D). We collected samples at 24h of growth similarly to Figure 3 and analyzed the cell morphology of the double A mutant by scanning electron microscopy. Growth (not shown) and morphology of the double A mutant were independent of addition of IPTG and similar to the wild type N6 strain, as well as the N6 pILL2150 control strain.

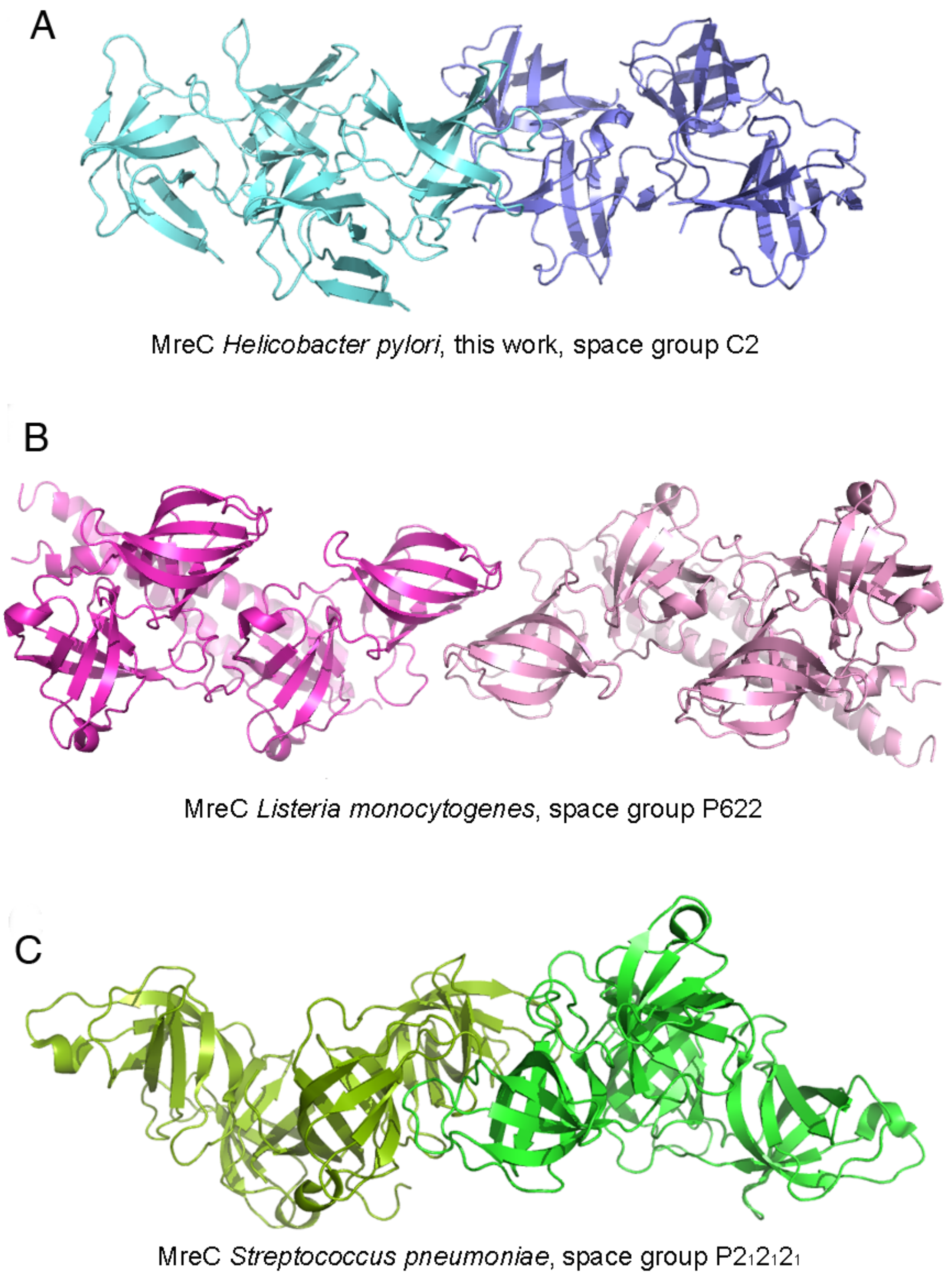

Supplementary Figure 6: Translational repeats within crystal structures of MreC variants indicate their self-association in reminiscent patterns. PDB codes for the different MreC variants are (A) *H. pylori*, 5LP5 (B) *L. monocytogenes*, 2J5U and (C) *S. pneumoniae*, 2QF4
